# Identifying the Dominant Personality Profiles in Medical Students: Implications for Their Well-Being and Resilience

**DOI:** 10.1371/journal.pone.0160028

**Published:** 2016-08-05

**Authors:** Diann S. Eley, Janni Leung, Barry A. Hong, Kevin M. Cloninger, C. Robert Cloninger

**Affiliations:** 1 School of Medicine, The University of Queensland, Brisbane, Australia; 2 School of Public Health, The University of Queensland, Brisbane, Australia; 3 Department of Psychiatry, School of Medicine, Washington University, St. Louis, Missouri, United States of America; 4 The Anthropedia Foundation, St. Louis, Missouri, United States of America; IRCCS Istituto Auxologico Italiano, ITALY

## Abstract

**Purpose:**

There is a high prevalence of stress, depression, and burn-out in medical students. Medical students differ widely in personality traits, self-perceptions, and values that may have an impact on their well-being. This study aimed to investigate variability in their personality profiles in relation to their potential for well-being and resilience.

**Method:**

Participants were 808 medical students from The University of Queensland. An online questionnaire collected socio-demographics and the Temperament and Character Inventory to assess personality traits. Latent profile analyses identified students’ trait profiles.

**Results:**

Two distinct personality profiles were identified. Profile 1 (“Resilient”) characterized 60% of the sample and was distinguished by low Harm Avoidance combined with very high Persistence, Self-Directedness and Cooperativeness compared to Profile 2 ("Conscientious"). Both Profiles had average levels of Reward Dependence and Novelty Seeking and low levels of Self-Transcendence. Profiles did not differ by age, gender, or country of birth, but rural background students were more likely to have Profile 1. While both Profiles indicate mature and healthy personalities, the combination of traits in Profile 1 is more strongly indicative of well-being and resilience.

**Conclusions:**

Finding two distinct profiles of personality highlights the importance of considering combinations of traits and how they may interact with medical students’ potential for well-being. Although both profiles of students show healthy personalities, many may lack the resilience to maintain well-being over years of medical training. Programs that develop character and personality self-awareness would enhance their well-being and prepare them to promote the health of their patients.

## Introduction

The high prevalence of stress, anxiety, burnout and depression among medical students is increasingly documented [1–4]. Hojat and colleagues have shown how humanistic components of clinical competence, such as empathy and other, interpersonal skills, can be eroded in medical students who are vulnerable to the rigors of medical training [5, 6]. There is growing awareness of threats to student health with many schools introducing or testing interventions and development programs to nurture and maintain overall well-being [7–10]. Such health promotion are important ways to increase the awareness of students who may be at risk of ill health.

Yet it is interesting to note that the well-being of some students is not and may never be under threat. In order to engage in effective health promotion for medical students, educators need answers to several key questions. Why is it that some students thrive during medical school whereas other flounder under stress? Could educators identify those at risk early in their education? Can the health of all medical students be enhanced so that they can develop the skills needed for lifelong well-being? In order to promote student health and well-being, educators need to recognize and consider how to help their students cultivate the configurations of personality traits that underlie positive health and resilience. Instilling such health awareness may not only benefit them as students, but help them understand how to promote the health of their patients, as physicians [11].

In April 2015 pre-medical students in the United States took the newest revision of the Medical College Admissions Test (MCAT) which emphasizes behavioral and social science, together with the traditional sciences [12]. Since 1928 when the first MCAT was introduced, medical educators have been concerned about assessing additional areas thought to be relevant to the education of physicians. Entry criteria in most schools are concerned with prior academic achievement and may include a range of cognitive indicators ranging from school grades to standardized aptitude tests, along with more qualitative measures such as interviews and references [13, 14]. While the traditional prerequisite of high academic standards cannot be disputed, many authorities are calling for consideration of the psychosocial characteristics of applicants because it takes more than high intellect to be a competent and ethical physician [15–20].

Contemporary issues in medical education seek to develop physicians who are not only intelligent but also compassionate and humanistic professionals. The physician of the future will be expected to be knowledgeable about how personal and social issues influence illness, as well as the influence of both biological and psychosocial processes on health and development. The practice of medicine will require the ability to communicate sensitively with people in diverse populations and communities. However, many contemporary efforts at improving medical education have not examined how well the varying personality profiles of different physicians match the demands of particular kinds of medical practice. What are the personality profiles observed in people who are attracted to and selected for the medical profession? How well are these personality profiles suited for a successful and satisfying career? How can medical educators help students to cultivate the full range of personal and social skills they need to practice medicine while maintaining their own health and promoting the health of their patients effectively?

Several personal competencies for medical students that are crucial to becoming a good physician have been proposed, but these are neither assessed nor considered in selecting students in most schools. For example, *inter-personal skills*, such as empathy, social warmth, and being cooperative and ethical, as well as *intra-personal skill*s such as being resourceful, purposeful, and responsible have been identified as important for effectiveness as a physician.[21] Such mature personal and social characteristics are also strongly associated the health and well-being of people in general,[22] and especially those who undertake challenging and demanding workloads, as do medical students [2]. The well-being of medical students can be severely affected by stressful situations, such as pressure for high achievement due to perfectionistic expectations from family, friends, and one's self, and practical concern about debt, while coping with psychosocial conflicts at the same time [19].

Personality is a strong predictor of well-being [23, 24]. However the effects of personality on well-being are complex, depending on combinations of multiple traits and the particular situation [1, 15, 19, 20]. Well-being may be defined differently by everyone as a result of each person’s unique experiences and interests, but all the components of health and wellbeing (i.e., physical, emotional, social, cognitive, and spiritual) are interdependent and influenced by personality [25].^.^ Research demonstrates how particular patterns of personality traits influence these components differently [26]. It has been shown that personality profiles with high Self-Directedness, Cooperativeness and Self-Transcendence and low Harm Avoidance are consistently most conducive to a mature personality and an individual’s positive perception of well-being, happiness and social support [27]. This is evident in general population samples [28] and in medical students [29]. This combination of traits is a strong predictor of someone being able to cope with life’s challenges, accept limitations, and develop strategies to bounce back from adversity [30].

Comprehensive indicators of the most desirable and relevant personal characteristics for medical students have been researched [20, 31]. This stems from the basic notion of educating students who are not just capable of successfully graduating from medical school, but those who are competent physically, mentally, and socially to practice over their lifetime. Research repeatedly calls for more consideration of the importance of personality and other non-cognitive characteristics in regards to student selection, counseling and support for student well-being [14, 20, 31, 32]. For example, Doherty and Nugent’s review [32] highlighted the trait of conscientiousness as a consistent predictor for academic success,[33, 34] clinical competence,[35, 36] and coping with stress [1, 15]. However, single traits have both costs and benefits dependent on the context [37]. Ferguson looked at the association of individual traits and learning outcomes across the medical program and illustrated how personality traits operate on a continuum within a given context [35, 37]. This paper aims to extend this notion and bring into focus the importance of looking at the combination of traits that need to be considered in the context of the whole person in multiple aspects of their life [25].

Most educators have exchanged anecdotal opinions suggesting that each new cohort of medical students is successively getting worse (or better) than the previous. However, it could be argued that medical students represent a rather homogenous population in many respects. Demographically their average age is 20–24. They are well-educated and often from a high socio-economic background. Their personalities are almost exclusively described as being intelligent, conscientious, confident, highly motivated and ambitious and perfectionist [18]. However, the literature highlights individual traits that often typify a certain behavioral trend of students. As noted above, traits operate in combination with other traits with effects that depend on the situational context [25, 37]. What is lacking in the literature on the implications of personality in medical students are comprehensive investigations of the effects of personality profiles (i.e., configurations of traits) on well-being. Looking at profiles of traits provides a more meaningful portrayal of an individual’s personality than studies of the same traits separately because it allows a better understanding of the various pathways to well-being and mature coping.

The objective of this study was to investigate whether there were distinct profiles or combinations of personality traits in a large sample of medical students. We assumed that despite the general homogeneity of medical student populations, there would be multiple profiles that portray their personalities. The aim was not to inform selection of medical students, although the data may provide insight into the combination of traits dominant in individuals who pursue medicine. The aim was to identify naturally occurring profiles of personality among medical students and to consider how these profiles may affect the student's ability to cope with the demands of medical training. Future publications will report empirical investigations of how profiles of personalities may influence measures of well-being.

## Methods

The study design was quantitative and cross-sectional using a self-report questionnaire to measure personality. Ethics was approved by the Behavioral and Social Sciences Ethical Review Committee at The University of Queensland. Participants provided written consent, which was documented on the questionnaire and approved by this ethics committee (Approval Numbers: 2013000495 and 2015001895).

### Participants and setting

Data were collected in 2014 and 2015 at The University of Queensland School of Medicine where entry is based only on grade point average and standardized test scores (GAMSAT: Graduate Medical School Admissions Test). In 2014 students in each year of the four-year medical program were asked to participate through a general invitation on the School’s student community website. It was discovered that this method did not reliably reach all students and therefore in 2015 we invited all Year 1 students to complete the questionnaire during a regularly scheduled activity. All students in both years were provided access to the identical questionnaire via an online link (Survey Monkey©).

### Measures

The questionnaire included basic socio-demographics (age, gender, country of birth, rural background and year of degree), and the Temperament and Character Inventory (TCI-R140) [38] to identify the seven basic dimensions of personality. The TCI is based on Cloninger’s psychobiological model of personality, which distinguishes between the personality domains of character traits (the cognitive domain of personality), and temperament traits (the emotional core of personality). The TCI is validated widely in more than 30 cultures on four continents. Its scales account for all components of alternative tests of personality, such as the five- and six-factor personality models [39], and is the strongest predictor of mature coping [40, 41]. The 140-item version uses a five point Likert scale (1 = absolutely false to 5 = absolutely true). Internal reliability (Cronbach alphas) of each dimension ranged from 0.86 to 0.89 for the character and 0.69 to 0.91 for temperament scales. Mean scores for each of the seven traits were used for the analysis.

The three character traits are Self-Directedness, Cooperativeness, and Self-Transcendence. *Self-Directedness* measures *intra-personal* character strengths, such as being responsible, purposeful, and admitting one’s own faults realistically. *Cooperativeness* measures *inter-personal* character strengths, such as tolerance, empathy, and respectful acceptance of the opinions and behaviors of others. *Self-Transcendence* measures *transpersonal* character strengths, such as being altruistic, idealistic, and self-forgetful, as occurs when people identify as an integral aspect of the world around them. It is often described as ‘inner spirituality’, as exemplified by people who are humble and accepting of their life versus someone who is conceited, materialistic and never satisfied. In brief, character traits are what we make of ourselves intentionally, so character is also the domain of personality that changes more rapidly in response to personal effort, social influences, and education [28, 42].

In contrast, temperament traits are more stable throughout life, but do respond to consistent behavioral conditioning [43]. *Novelty Seeking* measures an emotional drive to activate behavior because of curiosity to explore and to enjoy what is new and complex. *Harm Avoidance* measures an emotional drive to inhibit behavior because of a tendency toward anxiety and worry that anticipates problems and non-success. *Reward Dependence* measures an emotional drive to maintain behavior in response to cues of social reward, as is seen in people who exhibit sentimentality, social sensitivity, and dependence on approval by others. *Persistence* measures an emotional drive to anticipate reward and success, thereby allowing people to maintain behavior despite frustration or initial non-success, as is exhibited in people who are highly determined and ambitious [28]. High and low descriptors are summarized in Table 1.

**Table 1:** High and low descriptors for each temperament and character trait and average levels of each trait for the whole sample compared with population norms*.

Temperament traits	*Represents………*	LOW SCORES	*to* HIGH SCORES	Whole sample levels*
Novelty Seeking	*Exploratory activity in response to novelty*	*Orderly*, *reserved*, *rigid*, *thrifty*, *slow to anger*	*Exploratory*, *impulsive*, *extravagant*, *quick-tempered*	*Average*
Harm Avoidance	*Worry in anticipation of problems*	*Optimistic*, *accepting of uncertainty & risk*	*Pessimistic*, *fearful*, *shy*, *anxious*, *fatigable*	*Average*
Reward Dependence	*Dependence on approval of others*	*Critical*, *aloof*, *detached*, *not influenced by others*, *independent*	*Sentimental*, *open*, *warm*, *approval seeking*, *attached*, *dependent*	*High*
Persistence	*Industriousness of behaviour despite obstacles*	*Underachieving*, *flexible*, *easy-going*, *unambitious*	*Industrious*, *ambitious*, *determined*, *perfectionist*	*Very High*
**Character traits**	***Represents………***	**LOW SCORES**	***to* HIGH SCORES**	
Self-Directedness	*Responsibility*, *goal orientated & self-confidence*	*Blaming*, *aimless*, *helpless*, *defensive*, *conflicted*	*Responsible*, *purposeful*, *self-accepting*, *resourceful*, *self-actualising*	*Very High*
Cooperativeness	*Tolerance*, *cooperativeness & empathy*	*Prejudiced*, *insensitive*, *hostile*, *opportunistic*,	*Tolerant*, *empathic*, *helpful*, *forgiving*, *principled*	*Very High*
Self-Transcendence	*View of self in relation to the universe as a whole*	*Conventional*, *sceptical*, *pragmatic*, *materialistic*	*Self-forgetful*, *holistic*, *idealistic*, *spiritual*, *creative*	*Very Low*

Adapted from Cloninger et al, 1994 [38]

*Levels of each trait according to published population norms Cloninger 2006 [24]

### Analysis

To identify clusters of individuals with distinctive combinations of the seven traits, a latent profile analysis (LPA) was conducted on the trait scores using MPlus 6.12 [44].The LPA identified sub-groups (profiles) within the sample based on similarity of responses to the 7 trait scores. The analysis was conducted by comparing multiple profiles with a one-profile solution, adding more profiles until the additional one no longer yielded a significant improvement in model fit statistics. Determination of the number of profiles was based on a number of fit criteria. Good model fit indices were low Akaike information criterion (AIC), Bayesian information criterion (BIC) and sample size adjusted BIC. A lower value of these criteria indicates a better balance of model parsimony and model fit. Entropy was used to measure the classification uncertainty from 0 to 1. Higher values indicated clearer classification. A significant Lo-Mendell-Rubin likelihood ratio test (LMR-LRT) indicated that the current model had a significant better fit than the previous model (with one less profile). For descriptive purposes, t-tests compared the seven trait levels by the profiles identified from the LPA. Effect sizes were estimated using eta (η). Participants in each profile were compared by socio-demographics gathered. Using the auxiliary statement in MPlus, a three-step approach analysis based on the probability of classification was conducted to estimate odds of profile assignment by age, gender, country of birth, rural background, and year of medical school. Missing data were imputed using multiple imputation methods with the seven trait levels as the predictor, and the demographic variables for imputation only.

## Results

### Sample socio-demographics

The final sample of 808 represents two data collections as described above. The overall response rate was 62%. The mean age was 24.9 years, (55.9% under 25 years; range 19–59 years), 45.6% were female, 46.3% of students were born in Australia, and 21.3% reported a rural background. Year 1 participants accounted for 74.2%, with nearly equal proportions in Years 2–4 (Table 2).

**Table 2:** Socio-demographic characteristics of the whole sample of medical students (N = 808).

Survey year		n	%
	2014	339	42
	2015	469	58
**Age**			
	Mean (SD)	24.88	(4.00)
	Under 25 years	443	55.9
	25 or older	349	44.1
**Gender**			
	Male	439	54.4
	Female	368	45.6
**Country of birth**			
	Australia	372	46.3
	Not in Australia	432	53.7
**Rural background**			
	Yes	170	21.3
	No	630	78.8
**Year medical school**			
2015	Year 1	469	66.3
2014	Year 1	131	16.2
	Year 2	75	9.3
	Year 3	67	8.3
	Year 4	66	8.2

### Whole sample temperament and character levels

All scales were approximately normally distributed. Comparing the trait levels of the whole sample to percentile rankings representing population norms [24] showed these students are average in Novelty Seeking, average in Harm Avoidance, very high in Reward Dependence, Persistence, Self-Directedness and Cooperativeness, and very low in Self Transcendence.

### Trait profiles of medical students

Table 3 shows the LPA fit indices supported a two-profile solution. The LMR-LRT indicated that the two-profile model fit the data significantly better than the one profile model (p < 0.001) and not worse than a three profile model (p = 0.344). Entropy was also higher in the two-profile solution than it was in the three profile solution, which indicated that the two-profile solution had a clearer profile distinction. The BIC, AIC and LRT are presented in Table 3. The distribution of TCI scores of the LPA for three-profile solution is presented in Supporting Information (SI). We chose the two profile solution based on parsimony and greater entropy; the three-profile solution had lower entropy, only slightly lower BIC, and the LMR test was not significantly better. Description and follow-up tests for the two-profile solution are presented in Table 3.

**Table 3:** Fit statistics of latent profile analysis on the seven TCI trait scores establishing the two profile best fit.

	Profiles specified		
Fit statistics	1	2*	3
Log likelihood	-3978.77	-3701.06	-3635.50
Akaike information criterion	7985.54	7985.54	7331.00
Bayesian information criterion	8051.26	7549.39	7471.84
Sample size adjusted Bayesian information criterion	8006.81	7479.53	7376.57
Entropy	-	0.71	0.66
Lo-Mendell-Rubin likelihood ratio test	-	545.25	128.71
p-value	-	< .001	.344

Note:

*Model with best fit statistics as determined by marked lower AIC, BIC, SSABIC; highest Entropy, and a significant LMR-LRT p-value.

**Fig 1:**
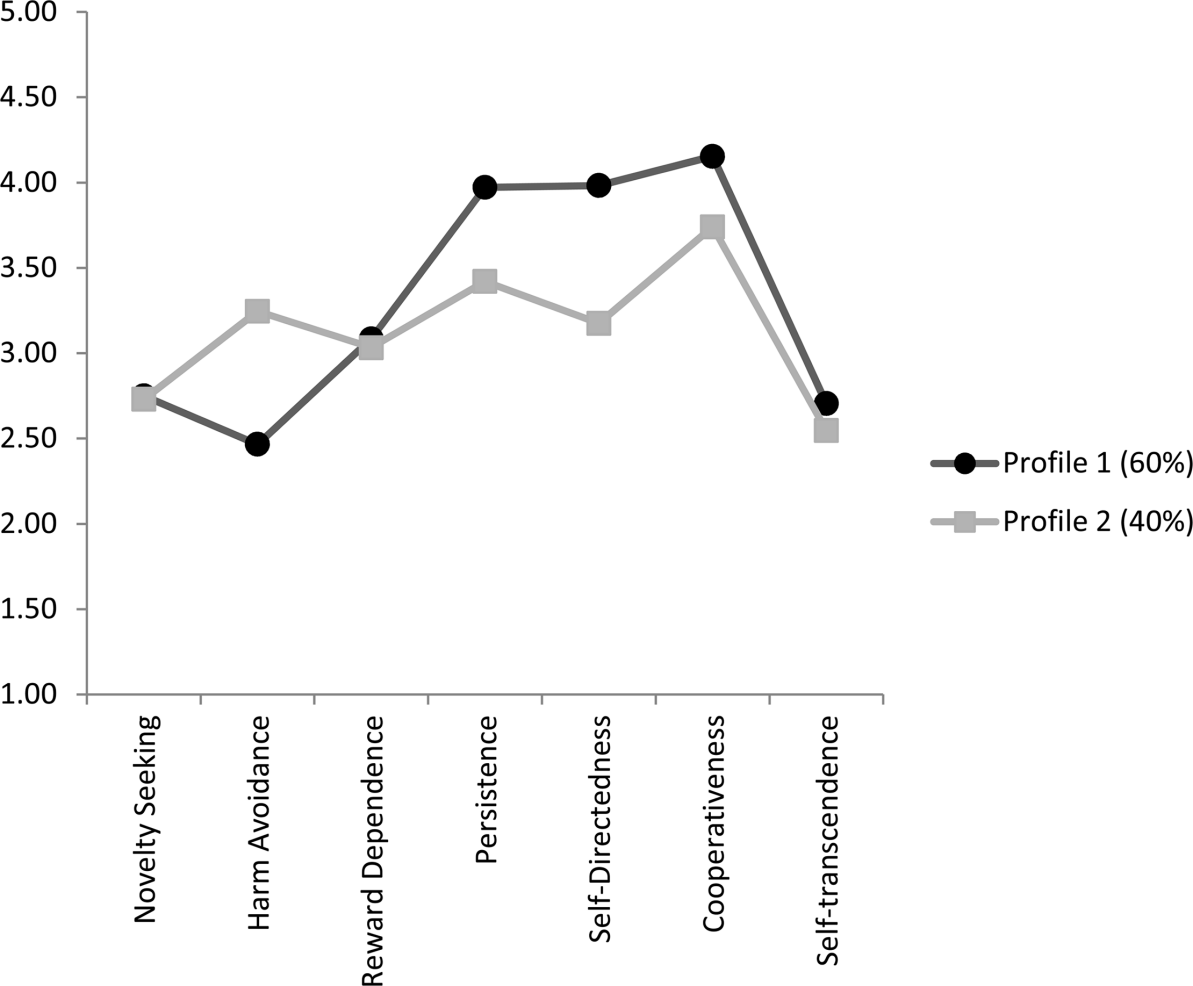
Graphic representation of the two temperament and character Profiles in medical students (N = 808).

The two profiles of medical students according to their TCI trait scores are presented in Fig 1 and Table 4. Sixty percent (n = 483) of students fell into Profile 1 and 40% (n = 325) into Profile 2. Profile 1 was characterized by lower levels of Harm Avoidance and higher levels of Persistence, Self-Directedness, Cooperativeness, Reward Dependence, and Self Transcendence compared to Profile 2, although effects sizes were weak for Reward Dependence and Self-Transcendence.

**Table 4:** The seven individual temperament and character traits by the two identified personality Profiles of medical students (N = 808).

	Profile 1		Profile 2		
	n = 483 (59.8%)		n = 325 (40.2%)		Statistics
Trait	Mean	(SD)	Mean	(SD)	T-tests^†^
Novelty seeking	2.75	(0.44)	2.74	(0.42)	t = 0.26; df = 806, p<0.796, η<0.01
Harm avoidance***	2.46	(0.57)	3.26	(0.53)	t = 20.47; df = 806, p<0.001, η = 0.59
Reward dependence**	3.09	(0.24)	3.03	(0.25)	t = 3.75; df = 806, p<0.01, η = 0.13^**+**^
Persistence***	3.98	(0.42)	3.40	(0.52)	t = 17.32; df = 806, p<0.001, η = 0.52
Self-Directedness***	3.99	(0.35)	3.15	(0.40)	t = 31.93; df = 806, p<0.001, η = 0.75
Cooperativeness***	4.16	(0.37)	3.72	(0.41)	t = 15.94; df = 806, p<0.001, η = 0.49
Self-Transcendence**	2.71	(0.72)	2.54	(0.69)	t = 3.32; df = 806, p<0.01, η = 0.11^**+**^

^†^Likert scale mean (1–5); standard deviation presented in parenthesis

*p<0.05

**p<0.01

*** p<0.001

^**+**^Note the negligible effect size for Reward Dependence and Self-Transcendence

Table 5 provides the odds ratios of medical students fitting in Profile 1, compared to Profile 2, by socio-demographic variables. Students from a rural background were at 2.34 higher odds (95%CI 1.44, 3.82) to fit in Profile 1. Year of medical school was significantly associated with trait profiles with students in later years of training less likely to fit in Profile 1. The trait profiles were not associated with age, gender, and country of birth.

**Table 5:** Odds ratios of medical students fitting in Profile 1 of the two identified personality Profiles by socio-demographic characteristics (N = 808).

	Odds ratio of fitting in Profile 1 ^†^(Reference is Profile 2)		

	OR	(95%CI)	p
**Age**			
Under 25 years	1.00	(Reference)	
25 or older	1.26	(0.87, 1.83)	0.215
**Gender**			
Male	1.00	(Reference)	
Female	1.24	(0.87, 1.78)	0.23
**Country of birth**			
Australia	1.00	(Reference)	
Not in Australia	1.39	(0.95, 2.03)	0.093
Rural background**			
Yes	2.34	(1.44, 3.82)	0.001
No	1.00	(Reference)	
Year medical school*			
Year 1	1.00	(Reference)	
Year 2	0.81	(0.44, 1.49)	0.499
Year 3	0.46	(0.24, 0.88)	0.019
Year 4	0.53	(0.28, 1.01)	0.054

^†^Profile 1 was characterized by lower levels of Harm Avoidance and, higher levels of Persistence, Self-Directedness, Cooperativeness, Reward Dependence, and Self Transcendence compared to Profile 2

## Discussion

This paper identified two distinct personality profiles in a large cohort of medical students. Students with personality Profile 1 are described as *resilient* because they combine being *vigorous* (high in Persistence and low in Harm Avoidance), *industrious* (high in both Persistence and Self-Directedness), and *versatile* (high in Self-Directedness and low in Harm Avoidance). Students with personality Profile 2 are referred to as *conscientious* and may be more anxious and less resilient than those with the resilient Profile 1, but both profiles are more mature, responsible, and well-organized than the average person in the general population. Profile 1 demonstrates a pattern of temperament and character that has previously been shown to predict greater life satisfaction and well-being, including its physical, mental, and social components [11, 20, 28].

The identification of only two distinct personality profiles is contrary to our expectation of finding multiple profiles among our sample. However, this two cluster solution illustrates the basic and key characteristics one would expect in medical students. It further reinforces the notion that these key characteristics, relevant to physicians in general, are related to the variability around overall resilience. Essentially, physicians need be fairly resilient with high Persistence plus high Self-Directedness and/or low Harm Avoidance. It is clinically interesting that the possible third profile that we considered identify a small sub-group of students who were less resilient. There could be a group of students who were more fragile in that they were higher in Harm Avoidance, lower in Persistence, and higher in Self-Directedness. Because there is no definitive statistical test of the optimal number of profiles to select, [45] it will be useful in future work to consider whether the two profile solution can be further validated by other academic and clinical measures, or whether more fine-grained descriptions are even more informative. Our current data indicate that the two-profile solution is a parsimonious and clear model that captures most of the variability in personality profiles among medical students.

Recent research has found five different personality clusters using the TCI in a study of physicians in a variety of specialties [45]. Nevertheless, our current report is the first study we know of that used a latent profile analysis to identify personality profiles based on a combination of personality traits in medical students. More research is needed to investigate whether students’ profiles become more differentiated as they gravitate toward certain specialties. This may suggest some development of traits in line with their interests but also may be the result of a better understanding of their personality and its fit with the certain specialties.

The only significant demographic association with either profile was that rural background students were more likely to fall into “resilient” Profile 1. This finding is congruent with previous work that has shown medical students of rural origin are more self-confident and resilient than their urban-origin counterparts [46–48]. Our findings contribute to the emerging notion of a rural-urban difference in adaptive personality profiles, and will be discussed in forthcoming publications.

We found a larger proportion of medical students in the first year of training fit the resilient profile more often than those in subsequent years. This finding may suggest that the experience of medical school is reducing the well-being of students rather than enhancing it. The literature has noted a decline in levels of empathy among third year students. [5] Although our data indicate that more first year students fit in the Resilient Profile, the study was not designed to investigate that trend. However, prospective studies are underway to follow this up over time to see if students’ profiles trend toward or away from being more resilient.

If it is the case that well-being deteriorates through medical school, students may benefit from psychological interventions that help enhance their well-being. Some evidence indicates that aspects of personality, such as Self-Directedness, Cooperativeness and Self-Transcendence can be increased through training in self-awareness or various mind-body exercises, which may lead to character development and enhanced well-being [49, 50]. In other words, medical training may provide a meaningful opportunity to help students to build character and strengthen their psychosocial weaknesses, thereby reducing their risk of burn-out and poor patient care. We propose that this is where personality testing has its primary and practical relevance in medical school—that is, helping students understand their personal trait profiles and further enhance the formation of their resilience, coping skills, and well-being.

### The implications of trait profiles to medical students and training

The overall levels of each trait in our sample when compared to population norms is similar to previous work where medical students and physicians consistently show higher levels of Persistence, Self-Directedness and Cooperativeness [47, 51, 52]. This combination of trait levels contributes to a profile that allows high achieving individuals to undertake a demanding profession such as medicine. It can be reasonably argued that few students could succeed in medical school unless they are highly persistent and self-directed. Likewise, to be highly cooperative is a trait that helps future physicians to engage in and maintain a myriad of professional and personal relationships, as is needed to perform successfully in their role. Individuals who are high in both Self-Directedness and Cooperativeness are consistently shown to demonstrate healthy personalities [22].^.^

Levels of Novelty Seeking among the whole cohort were found to be average in comparison to population norms. Average levels of Novelty Seeking among intelligent high achieving students suggest a balance between a tendency toward being too orderly and reserved and being too impulsive and extravagant. This very general depiction suggests a stable and sensible demeanor and is compatible with the overall maturity of medical students.

Our sample showed very high Reward Dependence in comparison to normative levels with only a small difference between profiles. Hence these students are likely to portray a high degree of social attachment and a pleasant, open manner beneficial to communication skills so vital to optimal patient care.

The average level of Harm Avoidance in this sample was similar to that observed in the general population. However, Harm Avoidance levels were lower among students in Profile 1 compared to Profile 2. Individuals low in Harm Avoidance tend to be calm, and tolerant of frustration whereas high levels indicate proneness to anxiety and a fear of uncertainty. These are important considerations in medical training and practice where stress and uncertainty are prevalent. Harm Avoidance is important to consider when examining trait profiles because the inclinations associated with high or low levels are influenced by the levels of other traits, in particular, very high Persistence and Self-Directedness.

Persistence is an important indicator of well-being because of its strong association with both perfectionism and a capacity to endure hard work and disappointment. Perfectionism is prevalent in high achieving individuals such as medical students and physicians [53, 54]. Few would doubt that perfectionism contributes to high quality control and is essential for all aspects of medical training and practice. However perfectionism exists in everyone on a continuum, with the one extreme being healthy and positive and the other self-defeating and negative [55, 56]. Therefore, high Persistence has both costs and benefits, and these dual aspects of Persistence are also influenced by interactions with levels of Harm Avoidance and Self-Directedness. Resilience is facilitated by combining high Persistence with low Harm Avoidance and high Self-Directedness, whereas susceptibility to stress and fatigue is greater when low Persistence is combined with high Harm Avoidance and/or low Self-Directedness [25]. These three traits comprise the components of a complex adaptive system, so their interactions have substantial influence on associated levels of resilience and perceptions of well-being [11]. Consequently, medical students with personality Profile 1 ("resilient") appears to have the healthier combination of traits because they have the synergistic combination of high Persistence, low Harm Avoidance, and high Self-Directedness in combination.

Certainly challenges encountered through medical training may influence one’s trait profile and students may gravitate toward a different profile dependent in part on how they cope. If we accept that students with the more resilient profile will cope better with challenges, then they may be at an advantage. However, it is most important for all students to be aware of their personal strengths and weaknesses in order to recognize a challenge and reflect on how to deal with it. In this way all students may enhance their resilience and well-being regardless of their initial personality profile.

While the two profiles of traits showed significant differences in the levels of Harm Avoidance, Persistence, Self-Directedness and Cooperativeness, it is of interest to note also the similarities between profiles in levels of Self-Transcendence. There is much in the literature around traits common among medical students and good reason to consider the generational implications of our data to students today. Twenge [18] discussed emerging trends in the behaviors of today’s medical students that suggest changes in personality, character, self-perception and values—all of which have an impact on well-being and ultimately progress in medical training. The current cohorts of medical students are primarily considered as Millennials, born 1981–1999, although there will likely be a smaller proportion of older students, or Generation Xers born 1965–1980. Research by Borges [57] found that Millennial students were significantly higher than Generation X in several traits including higher levels of perfectionism, feelings of entitlement and narcissism. The capacity to show empathy and compassion are arguably the two non-cognitive constructs of most interest for enhancement during medical school training.

Self-Transcendence pertains to an individual’s capacity for transpersonal identification; their capacity for trust, compassion, and altruistic behavior. The very low levels of Self-Transcendence among our sample in comparison to population norms suggest a tendency towards being practical, conventional and taking a skeptical evidence-based approach toward decisions. We saw that levels of Self-Transcendence did not differ between profiles, therefore in combination with high Self Directedness and Cooperativeness, our sample of students are portrayed as ‘organized’ characters who are typically leaders and successful because they are highly focused on their own interests and goals along with those of their close colleagues [27]. One might expect this to be an advantage to students pursuing the increasingly competitive environment of medicine. Nevertheless this generation is also characterized by several characteristics that are at odds with the increasingly competitive environment of medicine [18]. These circumstances could present challenges to individuals, not already high in resilience, to cope with setbacks or disappointment and may have implications for their capacity to show genuine compassion and humility as a doctor.

Increasing levels of Self-Transcendence in combination with already high Self-Directedness and Cooperativeness should foster patience, compassion, altruism, and tolerance of ambiguity [27]. This is an area that is worth developing amongst all medical students, in particular those who exhibit self-absorbed behaviors, insensitivity to the needs of others, and other signs of burn-out or distress, through interventions that promote the qualities of Self-Transcendence. There are many exemplar programs underway which mentor medical students to develop their self-awareness and professional identify through training which focuses on self-reflection, interpersonal communication skills and self-care. Instilling the realization of their responsibility for the privilege to help heal others requires training in compassion and equanimity. Our data suggest that all students might benefit from such training.

Our data support the rationale for the recent revision of the MCAT and its expansion in personal competencies, in particular because these competencies may be reflected in personality. However, our data is not about selection but about how to enhance personality formation once in medical school. Regardless of how they are selected, medical school is a demanding and stressful experience for most students, and many may encounter personal or academic challenges that will test their resilience. A non-stigmatizing person-centered approach, which provides information about how all medical students can personally work to promote their own health and cultivate greater character strengths, can provide a foundation for life-long well-being and a health-promoting professional identity [58]. The great need for physicians to be more effective in health promotion is shown by the relative ineffectiveness of current medical practice in reducing the burden of chronic non-communicable diseases, such as cardiovascular disease, chronic obstructive lung disease, cancer, diabetes, and depression [27].

Our study has some limitations. The design has self-selection bias due to the voluntary nature of participation especially in terms of the larger first year sample. First year students are generally more amenable to complying with requests to participate in extra activities. To help alleviate this possibility and ensure a more engaged and genuine response, a short seminar explaining this research is given beforehand, and the individual nature of their participation is emphasized by providing each student with their personal trait profile. A follow-up seminar is held to allow students to discuss the implications of their personal data, and the aggregate cohort data, in terms of the challenges they expect going forward in their medical training. Prior experience has shown the respondents to be genuine and engaged implying that the data will be relevant and valued. In addition, we conducted sensitivity analyses including only students in Year 1, and the same results were observed with the two identified profiles and their associated socio-demographic variables. Therefore, the sampling bias towards Year 1 students are unlikely to confound our findings. There is also the possibility that volunteers may bias their reports of personal traits to give a favorable impression of themselves. The data is descriptive so no causal influence can be assumed. The data are limited to one university in Australia, but similar personality findings have been found in students and physicians in the USA and Australia [51, 52]. Certainly our findings should be tested with other student populations and different personality measures. Our data is observational and does not imply cause and effect.

## Conclusions

The implications of this study go beyond simply identifying profiles of traits common to medical students. We hope our findings will help to motivate educators to assist medical students in developing greater self-awareness, which is the essential skill needed to strengthen their character and promote well-being. As noted by Epstein and Krasner [59] individuals must be able to understand their own “adaptive and maladaptive responses to stress” (page 301) before enacting strategies to address it.

In view of the importance of the health of medical students and physicians, how can educators enhance the resilience and well-being of otherwise bright, mature and high achieving students? We can and should strive to promote the health of the students and simultaneously prepare them to be effective in health promotion for their patients. To do so, educators need to change their perspective from focusing on getting problem students through medical training to an approach focused on ‘optimizing’ their health and well-being throughout medical school and beyond. Further work by the authors will investigate outcomes associated with these personality profiles in levels of resilience, tolerance of ambiguity and perfectionism.

## Supporting Information

S1 Dataset (XLSX)

S1 Text Distribution of TCI scores by 3 profile solution. (TIF)
