# Supplementary figures and images for: Identifying the Dominant Personality Profiles in Medical Students: Implications for Their Well-Being and Resilience
Source: PLoS One. 2016 Aug 5;11(8):e0160028. doi: 10.1371/journal.pone.0160028 (PMC4975484; doi:10.1371/journal.pone.0160028)

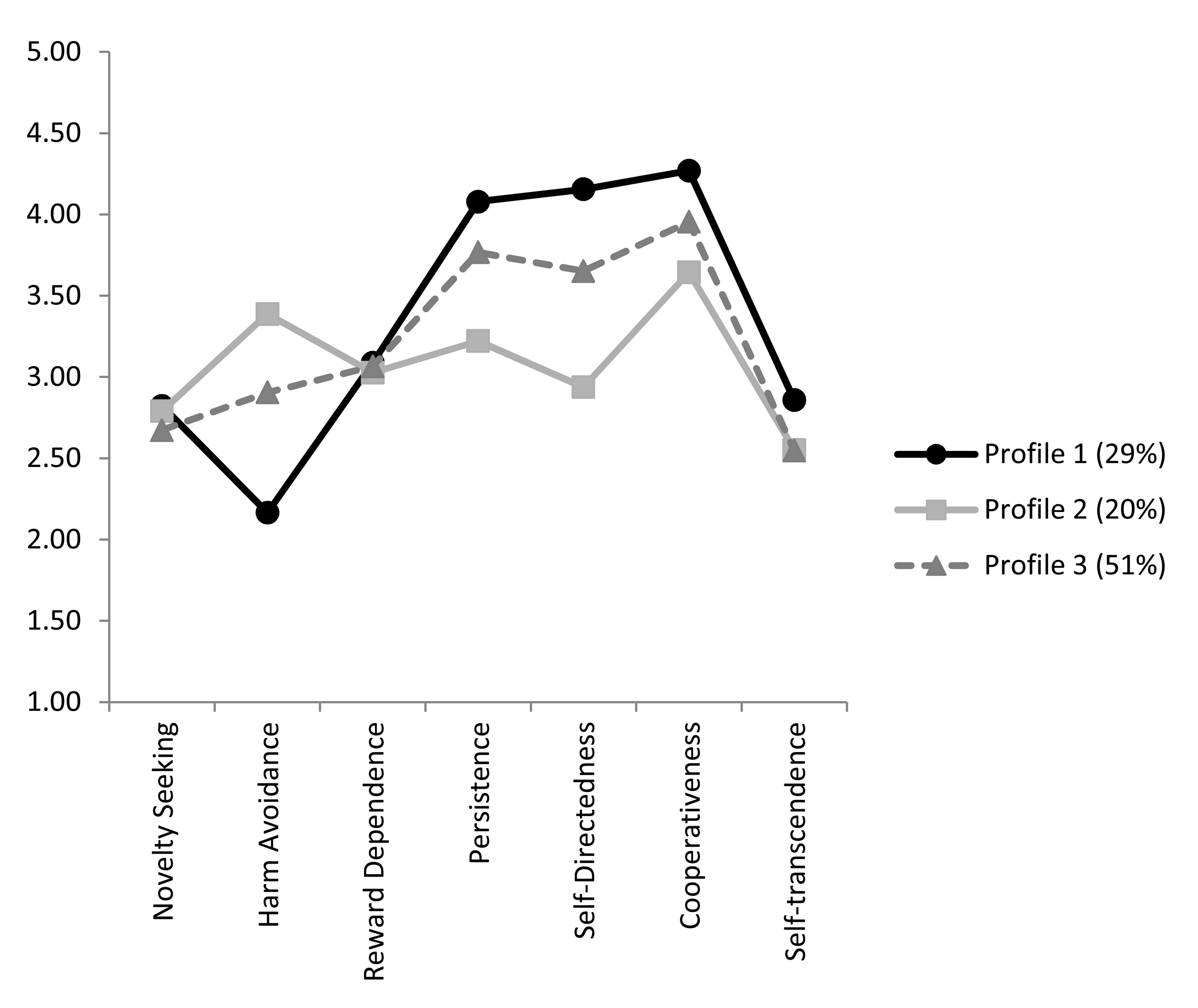

Supplement: S1 Text — (TIF) [file pone.0160028.s002.tif]
